# Real-world study to evaluate the efficacy and safety of intravitreal brolucizumab for refractory neovascular age-related macular degeneration

**DOI:** 10.1038/s41598-023-38173-y

**Published:** 2023-07-14

**Authors:** Hoseok Yeom, Hye Ji Kwon, Yoon Jeon Kim, Junyeop Lee, Young Hee Yoon, Joo Yong Lee

**Affiliations:** Department of Ophthalmology, Asan Medical Center, University of Ulsan College of Medicine, Seoul, Republic of Korea

**Keywords:** Diseases, Medical research

## Abstract

This retrospective study evaluated the real-world safety and effectiveness of switching to intravitreal brolucizumab for refractory neovascular age-related macular degeneration (nAMD). A total of 81 patients who received brolucizumab injections as switch therapy were followed for more than 3 months. A good response was defined as better anatomical improvement or extended injection intervals compared with previous anti-vascular endothelial growth factor (VEGF) treatment over a mean follow-up period of 41.4 weeks. Approximately 82.7% of patients showed a good response after switching. After 1 year, patients showed significant visual gains (+ 6.6 letters, *p* = 0.006) and central retinal thickness reductions (− 112.6 µm, *p* < 0.001), with 30.8% having injection intervals extended over 12 weeks. In the poor-response group, visual acuity and anatomical outcomes worsened soon after switching. More previous injections, thinner baseline central retina, and the presence of prechoroidal cleft or polypoidal lesion resulted in a better response (*p* < 0.05). Adverse effects occurred in eight eyes (9.9%), including one retinal vascular occlusion and seven intraocular inflammation cases, which were unrelated to the response. Most patients with nAMD refractory to anti-VEGF treatment demonstrated anatomical improvement or extended injection intervals after switching. This study shows that identified structural biomarkers may predict treatment response and select an appropriate therapeutic strategy.

## Introduction

Age-related macular degeneration (AMD) is the leading cause of irreversible blindness in people aged ≥ 50 years^1^. Anti-vascular endothelial growth factor (VEGF) agents are widely used for treating neovascular AMD via intravitreal injection with studies reporting reductions in the incidence of blindness of up to 50%^2,3^. However, some neovascular AMD cases do not respond to anti-VEGF treatment or the treatment gradually becomes less effective over time. Studies are still ongoing to develop new durable and long-lasting treatments to alleviate the burden of managing neovascular AMD^4^.

The goal of neovascular AMD treatment is to safely minimize disease activity, such as fluid collection, and improve visual outcomes^5^. Brolucizumab (Beovu^®^, Novartis, Basel, Switzerland) is the newest anti-VEGF agent approved for treating neovascular AMD. This new, small molecule (26 KDa), with a high concentration of 6.0 mg and a high binding affinity for multiple isoforms of VEGF-A, can increase the durability of the treatment response^6^. The HAWK and HARRIER studies demonstrated the non-inferiority of brolucizumab to aflibercept, and showed better anatomic results and prolonged duration of effect^7^. In a well-controlled study, improvements in both visual acuity and retinal thickness were reported after switching to brolucizumab 1 month later^8^. However, there is limited knowledge regarding long-term evaluation after switching to brolucizumab in refractory neovascular AMD. Specifically, it is unclear which patients respond well after switching, and whether injection intervals could be extended further. Therefore, this study aimed to analyze the real-world efficacy and safety of intravitreal brolucizumab injections as a switch therapy.

## Methods

This retrospective study included patients with a defined ophthalmological status who received intravitreal brolucizumab injections to treat neovascular AMD at the Seoul Asan Medical Center between July 2021 and December 2022. This study was approved by the Institutional Review Board of Asan Medical Center (IRB FILE No. 2022-0978) and the need for informed consent was waived due to the retrospective chart review study. The study adhered to the tenets of the Declaration of Helsinki.

### Patients

The inclusion criteria were patients with refractory neovascular AMD aged ≥ 50 years who received two or more injections of brolucizumab as switch therapy and were followed up for more than 3 months. In cases where switching was deemed necessary to reduce treatment burden, the definition of refractory neovascular AMD was determined based on the clinical judgment of the physician. This definition not only included cases where there was a poor response despite maximum treatment but also cases where there was no response to the previous treatment or a diminishing response over time, indicating resistance. The patients received their first intravitreal brolucizumab injection at least 2 months after treatment with another anti-VEGF agent (bevacizumab, ranibizumab, or aflibercept) according to the treat-and-extend protocol developed by four retina specialists (Y.J.K., Y.L., Y.H.Y., and J.Y.L.). All the specialists treated neovascular AMD with extended injection intervals of at least 8 weeks, usually in the presence of a dry macula. Two to four weeks after the first brolucizumab injection, both the response and intraocular inflammation were evaluated. Patients with neovascular AMD can be classified into three subtypes according to the type of neovascular AMD: type 1/2/mixed macular neovascularization (MNV), polypoidal choroidal vasculopathy (PCV), or type 3 MNV (retinal angiomatous proliferation [RAP]). Before the study, the patients’ medical histories were reviewed and patients with one or more of the following criteria were excluded: history of intraocular surgery during injection treatment, active proliferative diabetic retinopathy, vitreous hemorrhage, previous treatment without intravitreal anti-VEGF injections (e.g., focal laser, photodynamic therapy, or vitrectomy), or any history of coexisting pathology that could cause macular edema, such as retinal vascular disease, ocular inflammatory disease, epiretinal membrane or vitreo-macular traction.

### Data collection

All patients underwent a standard ophthalmologic examination comprising visual acuity (VA), fluorescein (FA) and indocyanine green angiographies (ICGA), and optical coherence tomography (OCT; Spectralis^®^ HRA + OCT, Heidelberg Engineering, Heidelberg, Germany). VA was converted to the Early Treatment Diabetic Retinopathy Study (ETDRS) letter score for analysis. The conversion can only be considered as an approximation because of the inclusion of protocol refraction in ETDRS acuity^9–11^.

Central macular thickness (CRT) was measured using the built-in OCT software. Subfoveal choroidal thickness (SCT) and pigment epithelial detachment (PED) were measured manually by two independent ophthalmologists (H.Y. and H.J.K.). The PED tracked the highest lesion change in the 9 × 9 mm area of the fovea at the first injection. For each parameter, an average of two measurements was used for the analysis. The presence of polypoidal lesions was evaluated on OCT findings (sharply peaked PED) and correlated with ICGA findings (polyp-like choroidal vessel dilation)^12^. Other structural OCT biomarkers, such as prechoroidal cleft and hyper-reflective dots, were considered to be predictive factors of treatment response^13^.

The patients were classified as either “good” or “poor” responders by two independent ophthalmologists (H.Y. and H.J.K.). In case of disagreements, a third ophthalmologist (J.Y.L.) acted as adjudicator. The good-response group included patients who showed better anatomical improvement or received injections at more extended intervals compared with previous anti-VEGF treatment. A good anatomical outcome refers to a noticeable improvement in retinal structure resulting from the reduction of subretinal fluid, intraretinal fluid, or PED. Conversely, the poor-response group included patients who experienced similar or worse outcomes than their previous treatment.

The state of fluid was compared with their previous visit and classified into four types at each visit. “No fluid” was defined as dry-up with no subretinal or intraretinal fluid at the time of the visit. If less fluid was present than the previous visit, it was set as “reduced but persistent fluid.” Cases in which fluid amount was similar or worse compared with the previous visit were defined as either “stable fluid” or “increased fluid,” respectively.

### Statistical analysis

Statistical calculations were performed using Statistical Package for Social Sciences Version 20.0 (SPSS Inc., Chicago, USA). Percentage, mean, and standard deviation were calculated for all quantitative variables. Intergroup comparisons were evaluated using the independent *t*-test and the paired *t*-test, while the Pearson Chi-squared and Fisher’s exact tests were used to analyze the association between variables. p-values < 0.05 were considered statistically significant. To assess the association between the OCT findings and good/poor responses, the odds ratio (OR) was determined using a standard formula. The calculation approach followed the conventional method of case/control and exposed/unexposed, representing the odds of exposure in cases compared to controls. For a precise estimation of the OR, the same SPSS statistical software package was used, and appropriate statistical tests were conducted. The significance level was also set at a predefined alpha level (p < 0.05).

## Results

### Demographic characteristics

A total of 81 patients (81 eyes) diagnosed with neovascular AMD underwent intravitreal brolucizumab injections as switch therapy. Altogether 67 patients (82.7%) showed a good response after switching, while the remaining 14 patients (17.3%) had a poor response (Table 1). All patients were of Asian ethnicity.

**Table 1:** Demographic and clinical characteristics of the study population.

Characteristics	Total	Good-response group	Poor-response group	*p*-value
Number of eyes, n (%)	81	67 (82.7%)	14 (17.3%)	
Age (years)	70.6 ± 6.6 [56–85]	70.3 ± 6.7 [56–85]	72.1 ± 6.1 [59–80]	0.351*
Sex, male (%)	55 (67.9%)	46 (68.7%)	9 (64.3%)	0.485^†^
Systemic disease				
HTN, n (%)	46 (56.8%)	40 (59.7%)	6 (42.9%)	0.253^†^
DM, n (%)	14 (17.3%)	12 (17.9%)	2 (14.3%)	0.748^†^
Type of AMD				0.073^†^
Type 1/2/mixed MNV, n (%)	33 (40.7%)	24 (35.8%)	9 (64.3%)	
PCV, n (%)	37 (45.7%)	33 (49.3%)	4 (28.6%)	
Type 3 MNV, n (%)	11 (13.6%)	10 (14.9%)	1 (7.14%)	
Baseline VA (letters)	56.7 ± 18.7	56.4 ± 18.0	58.3 ± 25.6	0.733*
Baseline CRT (µm)	380 ± 135	356 ± 118	496 ± 155	0.006*
Baseline SCT (µm)	271 ± 118	266 ± 116	295 ± 128	0.404*
Baseline PED (µm)	250 ± 162	255 ± 156	226 ± 196	0.543*

*HTN* hypertension, *DM* diabetes mellitus, *MNV* macular neovascularization, *VA* visual acuity, *CRT* central retinal thickness, *SCT* subfoveal choroidal thickness, *PED* pigment epithelial detachment. p-values for the difference between the good and poor responder groups were obtained. *Independent *t*-test; ^†^Pearson Chi-squared test. Significant values are in bold.

The mean age of the patients was 70.6 ± 6.6 years (range 56–85). Fifty-five patients (67.9%; mean age, 71.2 ± 6.0 years) were male and 26 (32.1%; mean age, 69.4 ± 7.7 years) were female. Forty-six patients (56.8%) had hypertension and 14 patients (17.3%) had diabetes mellitus. Differences in age, sex, and underlying disease were not related to treatment response after switching (Table 1). Thirty-three patients (40.7%) were diagnosed with type 1/2/mixed MNV, 37 (45.7%) with PCV, and 11 (13.6%) with type 3 MNV. In the good-response group, PCV was the most common (33 eyes, 49.3%), while 64.3% of patients in the poor-response group (nine eyes) had type 3 MNV. The treatment response after switching to brolucizumab was not significantly associated with the subtype (*p* = 0.073).

### Anti-VEGF treatment

All eyes had previously been receiving anti-VEGF injections (bevacizumab, ranibizumab, or aflibercept). Before switching to brolucizumab, all 81 eyes received an average of 24.5 anti-VEGF injections at 7.5-week intervals. The good-response group had received significantly more previous injections than the poor-response group (25.8 vs. 18.7, *p* = 0.034, Table 2). The intervals between previous injections were not associated with the response after switching (7.5 vs. 7.3, *p* = 0.641). Following the first brolucizumab injection, the mean follow-up period was 41.4 weeks. During this period, an average of 5.2 intravitreal brolucizumab injections were administered at 10.5-week intervals. After switching, the mean injection interval was extended by more than 3 weeks (*p* < 0.001). The mean injection interval was also extended in the poor-response group, but it was not statistically significant (*p* = 0.187). In 25 eyes (30.9%), the interval could be extended to 12 weeks or more after switching to brolucizumab, and the intervals were significantly longer in the good-response group compared with the poor-response group (*p* = 0.028).

**Table 2:** Treatment response to intravitreal brolucizumab injection in patients with refractory neovascular AMD.

Characteristics	Total (N = 81)	Good-response group (N = 67)	Poor-response group (N = 14)	*p*-value
Anti-VEGF injection				
Previous injections before the switch (n)	24.5 ± 15.2 [3–72]	25.8 ± 15.9	18.7 ± 9.4	0.034*
Number of anti-VEGF agents taken before (n)				0.260^†^
1 agent	32	29	3	
2 agents	42	32	10	
3 agents	7	6	1	
Total brolucizumab injections (n)	5.2 ± 2.4 [2–21]	5.3 ± 2.5	4.6 ± 1.5	0.295*
Follow-up period after the switch (weeks)	41.4 ± 14.5 [12–68]	42.9 ± 13.9	34.5 ± 15.7	0.049*
Interval of previous injection (weeks)	7.5 ± 2.1 [4.0–12.5]	7.5 ± 1.9	7.3 ± 2.6	0.641*
Interval of brolucizumab injection (weeks)	10.5 ± 2.5 [8.0–25.6]	10.8 ± 2.8	9.1 ± 1.1	0.028*
	*p* < 0.001**	*p* < 0.001**	*p* = 0.187**	
OCT biomarkers				
Pre-choroidal cleft, n (%)	26 (32.1%)	25 (37.3%)	1 (7.14%)	**0.030**^ǂ^
Polypoidal lesion (sharply peaked PED), n (%, PCV)	31 (83.8%)	30 (90.9%)	1 (25.0%)	**0.008**^†^
Hyper-reflective dots, n (%)	56 (69.1%)	44 (65.7%)	12 (85.7%)	0.206^ǂ^

p-values for the difference between the good and poor responder groups were obtained. *Independent *t*-test; **Paired *t*-test; ^†^Pearson Chi-squared test; ^ǂ^Fisher’s exact test. Significant values are in bold.

### Visual acuity and OCT findings

**Figure 1:**
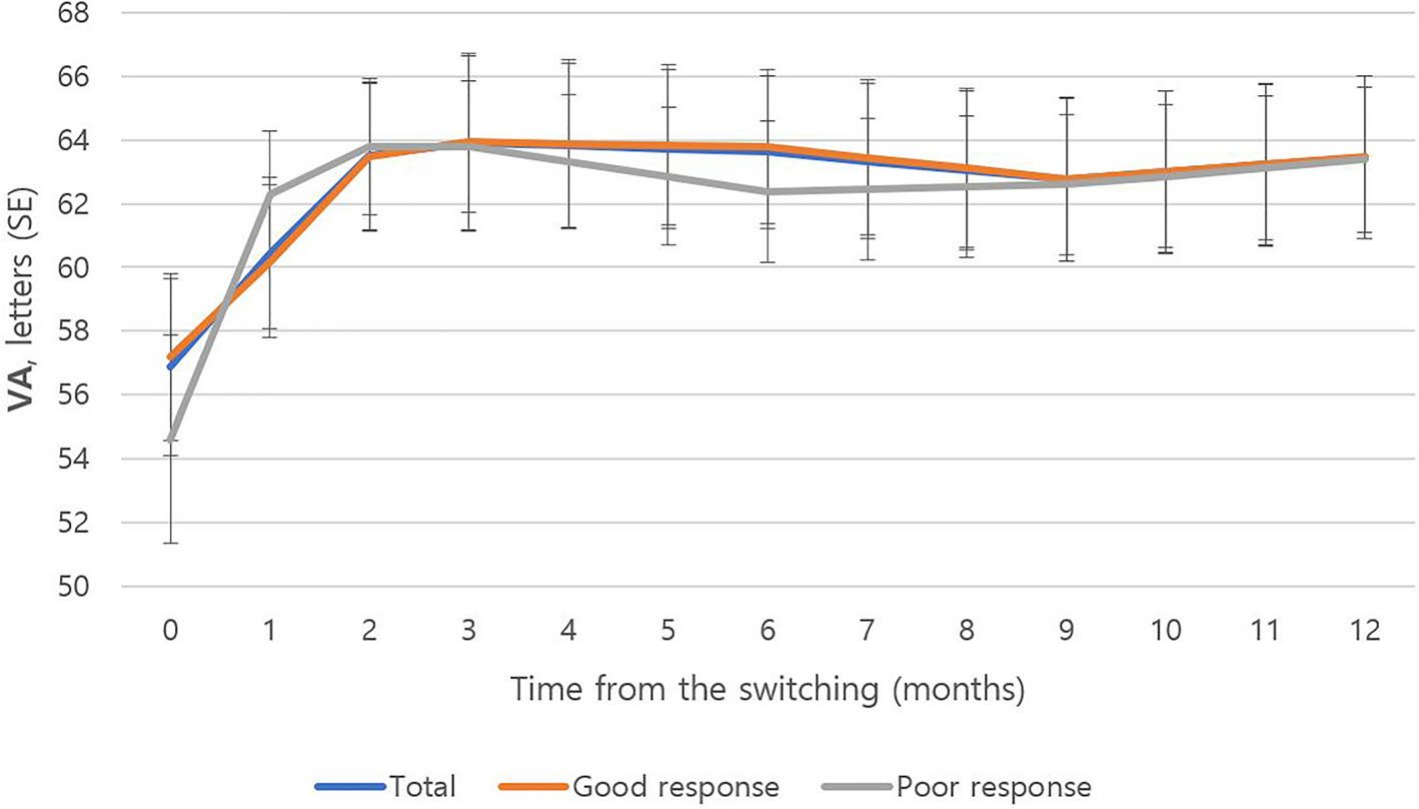
One-year changes in visual acuity (VA) after switching to brolucizumab (N = 40). *SE* standard error.

The mean VA of all patients (81 eyes) at baseline was 56.7 ± 18.7 letters (Table 1). The mean VA at baseline was 56.4 ± 18.0 letters in the good-response group (67 eyes), and 58.3 ± 25.6 letters in the poor-response group (14 eyes). There was no significant difference between the two groups (*p* = 0.733). Among the 40 eyes who had more than 1 year of follow-up, the initial mean VA was 56.9 ± 18.0 letters (Fig. 1). The mean VA 1 year after switching to brolucizumab was 63.5 ± 16.6 letters, and the mean letter gain was + 6.6 letters (*p* = 0.006). Among them, in the good-response group (35 eyes), the initial mean VA was 57.2 ± 17.1 letters, and the last mean VA was 63.5 ± 16.7 letters 1 year after switching. The mean letter gain was + 6.3 letters in this group (*p* = 0.003). In the poor-response group (five eyes), the mean VA improved by + 8.8 letters from 54.6 ± 25.4 to 63.4 ± 17.8 letters during the 1-year follow-up, although this was not statistically significant (*p* = 0.089).

**Figure 2:**
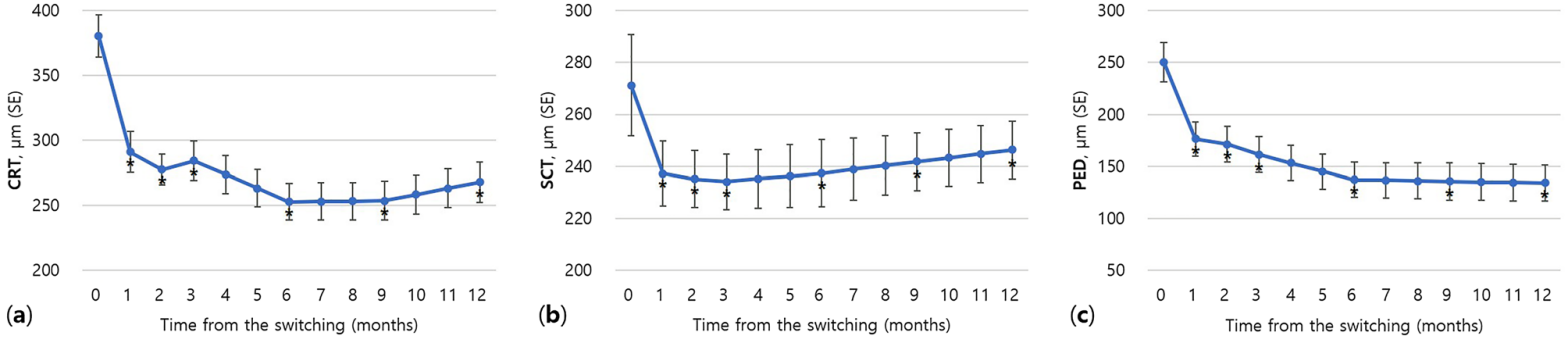
One-year changes in (**a**) central retinal thickness (CRT), (**b**) subfoveal choroidal thickness (SCT), and (**c**) pigment epithelial detachment (PED) after switching to brolucizumab (N = 40). *Decreased significantly compared with baseline (*p* < 0.001); *SE* standard error.

**Figure 3:**
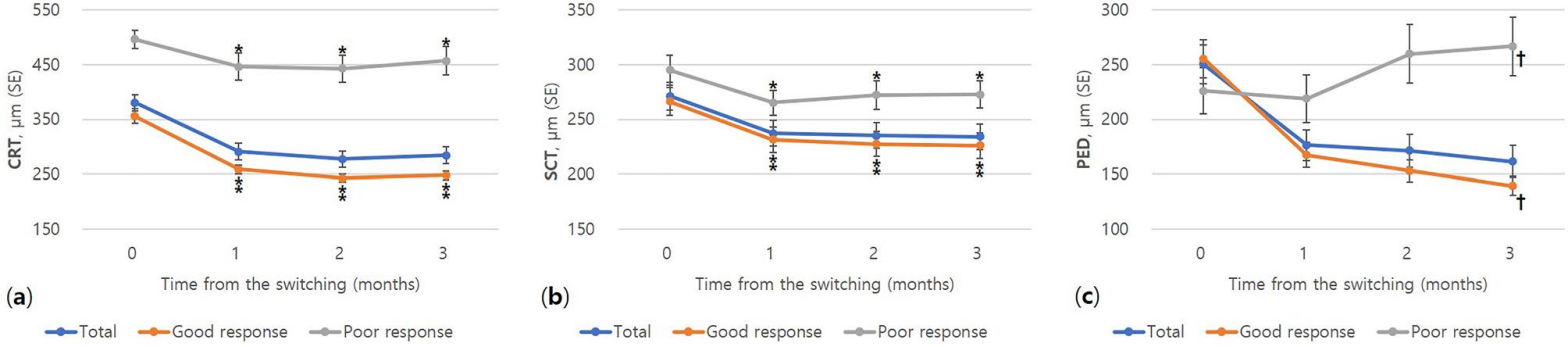
Early changes in (**a**) central retinal thickness (CRT), (**b**) subfoveal choroidal thickness (SCT), and (**c**) pigment epithelial detachment (PED) stratified by treatment response after switching to brolucizumab (N = 81). *^,#^Decreased significantly compared with baseline (*p < 0.01, ^#^*p* < 0.05); ^**†**^Significant difference between good and poor-response groups (*p* < 0.05); *SE* standard error.

The mean baseline CRT for all patients (81 eyes) was 380 ± 135 µm (Table 1). The mean CRT at baseline was 356 ± 118 µm in the good-response group (67 eyes), and 496 ± 155 µm in the poor-response group (14 eyes). A significant difference was observed in the baseline CRT between the two groups (*p* = 0.006). In 40 patients that were followed up for more than 1 year, the CRT decreased significantly from 382 ± 105 µm before the initial switching treatment to 268 ± 99 µm after 1 year (− 112.6 µm, *p* < 0.001, Fig. 2a). The SCT decreased significantly from 282 ± 124 µm before the initial treatment to 246 ± 113 µm after year (− 24.9 µm, *p* < 0.001, Fig. 2b). The height of PED decreased significantly from 222 ± 126 µm before the initial treatment to 134 ± 72 µm after 1 year (− 116.0 µm, *p* < 0.001, Fig. 2c). In all 81 eyes, initial changes in the 3 months after switching to brolucizumab were compared by treatment response group (Fig. 3). In the first month after the initial injection, anatomical improvements were observed in both patient groups (81 eyes, *p* < 0.001). However, in the poor-response group, the SCT decreased after the first month and continued to worsen for the next 2 months (14 eyes, *p* < 0.05, Fig. 3b). After 3 months, the height of PED showed a significant difference between both groups in treatment response (139 vs. 267 µm, *p* = 0.044, Fig. 3c).

Structural OCT biomarkers were identified before the initial injection and analyzed to predict treatment response after switching to brolucizumab (Table 2). Prechoroidal cleft was identified in 26 eyes (32.1%) in total, of which 25 of the 26 eyes were in the good-response group. The treatment response after switching was significantly better when the prechoroidal cleft was identified (odds ratio [OR] = 7.74, *p* = 0.030). Sharply peaked PED was identified in 31 (83.8%) out of 37 eyes with PCV, of which 30 of the 31 eyes had good-response PCV (33 eyes) and one eye had poor-response PCV. There was a significant association between sharply peaked PED and a good response to brolucizumab after switching (OR = 10.54, *p* = 0.008). Hyper-reflective dots were identified in 56 eyes (69.1%) in total, 44 of the 56 eyes in the good-response group, and 12 eyes in the poor-response group. There was no statistically significant association between hyper-reflective dots and treatment response (*p* = 0.206).

### Fluid status

**Figure 4:**
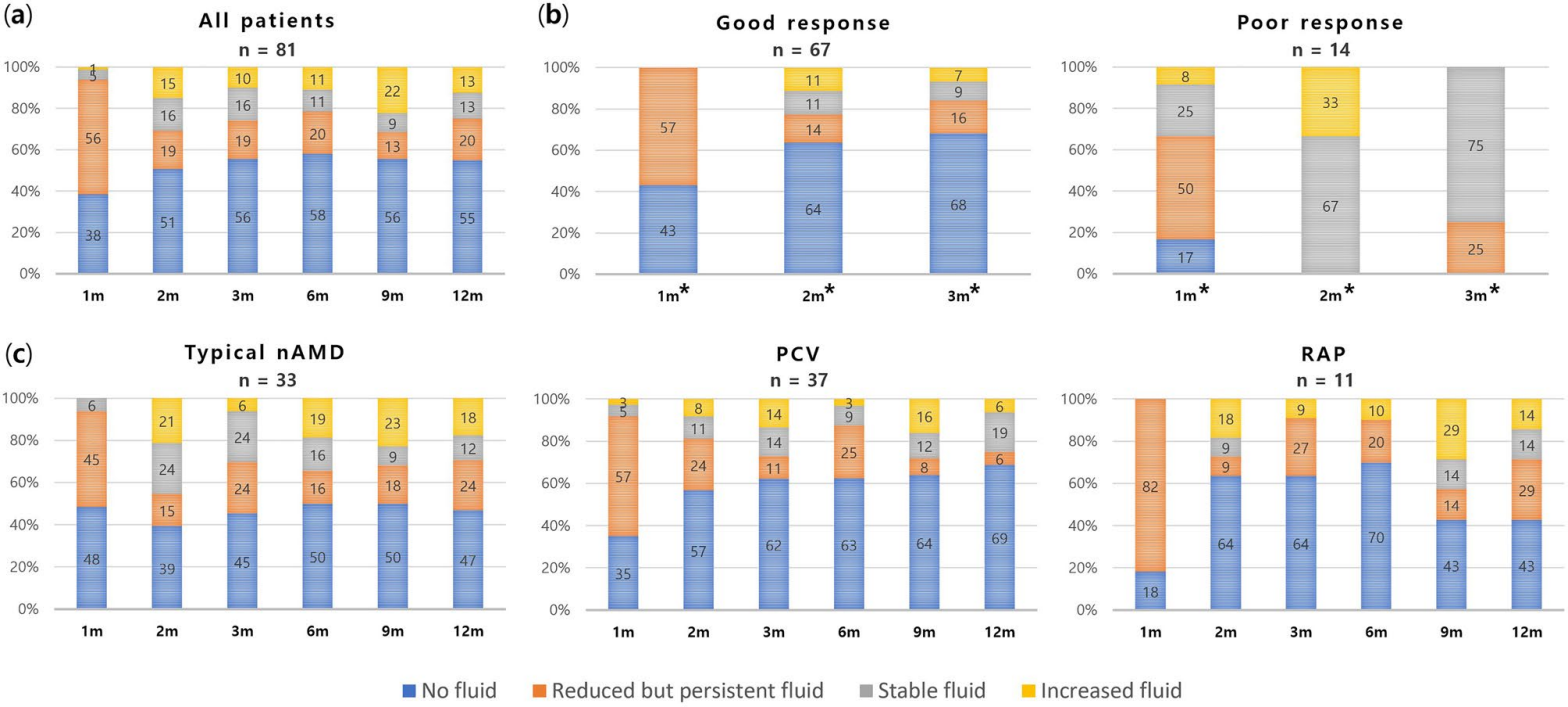
(**a**) One-year changes in the status of fluid. (**b**) Early changes in the status of fluid by treatment response. *Significant difference among the visit (*p* < 0.01). (**c**) One-year changes in the status of fluid by subtypes of neovascular AMD.

One-year changes in the subjective grading of fluid after switching to brolucizumab are shown in Fig. 4a. Fluid was reduced in more than 90% (76 eyes) of patients within the first month. Approximately 38% (31 eyes) of patients got dry macula after a single brolucizumab injection. The rate of complete resolution of fluid increased from 2 to 6 months after switching and lasted for 1 year in more than half of the patients. Early fluid changes were compared by dividing the group by treatment response (Fig. 4b). In the good-response group, fluid amount was reduced in all patients within the first month, with no fluid decrease in the first month for the poor-response group. There was also no decrease in fluid compared with the previous visit after 2 months and 4 months. Patients who achieved complete resolution of fluid at this point were all in the good-response group. One-year changes were compared by the subtypes of neovascular AMD (Fig. 4c). The rate of complete resolution of fluid in the first month was higher in type 1/2/mixed MNV and PCV than that in type 3 MNV, which showed fluid improvement in all patients in the first month. The highest rate of dry macula persistence at 1 year was found in patients with PCV.

### Adverse events

None of the eyes developed systemic adverse events after switching to brolucizumab. However, eight eyes (9.9%) were shown to have ocular adverse events associated with brolucizumab (Table 3). Seven of these patients (87.5% of eight adverse events) discontinued brolucizumab and switched back to previous anti-VEGF agents, while one patient who had a mild adverse event remained on brolucizumab. Intraocular inflammation presented with either anterior segment cells (two eyes, 2.5%) or vitreous opacity (five eyes, 6.2%). Retinal vascular occlusion was noted in one eye (1.2%) with a good response after switching. Treatment included topical corticosteroid eye drops (two eyes), oral and topical corticosteroid (three eyes), a topical corticosteroid with subtenon triamcinolone acetonide (one eye), and oral and topical corticosteroid with subtenon triamcinolone acetonide (two eyes). All the eyes improved without any complications after treatment, except for one retinal vascular occlusion case that experienced mild visual field defect sequelae. No significant association was observed between adverse events and the treatment response after switching to brolucizumab (*p* = 0.621).

**Table 3:** Adverse events of brolucizumab.

Characteristics	Total (N = 81)	Good-response group (N = 67)	Poor-response group (N = 14)	p-value
Switch back, n (%)	12 (14.8%)	7 (10.4%)	5 (35.7%)	**0.029**^ǂ^
Cause				
Adverse events, n (%)		5 (7.46%)	2 (14.3%)	
Low response, n (%)		–	3 (21.4%)	
Other, n (%)		2 (2.99%)	–	
Adverse events, n (%)	8 (9.88%)	6 (8.96%)	2 (14.3%)	0.621^ǂ^
Anterior uveitis, n (%)	2 (2.47%)	1 (1.49%)	1 (7.14%)	
Vitreous opacity, n (%)	5 (6.17%)	4 (5.97%)	1 (7.14%)	
Retinal vascular occlusion, n (%)	1 (1.23%)	1 (1.49%)	-	

p-values for the difference between the good and poor responder groups were obtained. ^ǂ^Fisher’s exact test. Significant values are in bold.

## Discussion

In this study, most of the patients who failed to respond to routine treatment showed excellent anatomical and visual outcomes compared with previous anti-VEGF treatments. Additionally, the injection interval could be extended after switching to brolucizumab. The goal of treating neovascular AMD is to reduce disease activity and improve visual outcomes. However, to reduce the overall burden of treatment, more durable and long-lasting treatment is needed. Studies have shown that brolucizumab is a potent intravitreal anti-VEGF agent with similar safety and efficacy to those currently used to treat neovascular AMD^14,15^. Additionally, brolucizumab may be a long-lasting drug, primarily because of its low molecular weight, allowing for high molar doses^16^.

In the HAWK and HARRIER protocols (brolucizumab 6.0 mg), more than half of the patients with neovascular AMD and 76% of patients with PCV were successfully maintained on a q12w interval for 48 weeks^7^. In the current study, only 30.8% (25 eyes) of patients extended the injection interval to 12 weeks for 1 year. The fact that the patients in this study were refractory to previous anti-VEGF treatment might be the reason for this difference. The 12-week injection interval was the longest among the treat-and-extend protocol used worldwide. The high affinity for VEGF and low molecular weight of brolucizumab allow for more drug delivery per injection than other anti-VEGF agents and offer the potential for effective penetration and durable action^17^. This might reduce the overall treatment burden for patients with neovascular AMD.

Statistically significant increases in mean VA and decreases in mean CRT, SCT, and PED were identified from baseline to the final visit. The mean visual gain was + 6.6 letters at the 1-year follow-up (*p* = 0.006), which was similar to the HAWK (+ 6.6 letters, *p* < 0.001) and HARRIER (+ 6.9 letters, *p* < 0.001) trials^7^. A recent study on real-world evidence with brolucizumab observed significant visual improvement at the final follow-up. The REBA (+ 10.4 letters, *p* = 0.014) and BRAILLE (approximately + 10 letters, *p* < 0.00001) studies reported large visual gains^18,19^. In contrast, Enriquez et al., SHIFT, BREW studies, and Giunta et al. found no significant VA improvements^20–23^. Therefore, a “ceiling effect” may explain the difference in visual gain when baseline VAs are different^24^. Haensli et al. reported improved reading acuity of approximately 0.32 to 0.5 Snellen decimal after 6 months using a standard reading chart^25^.

Few studies have reported on the change in SCT. In this study, SCT decreased significantly after 1 month, reaching its lowest point at 3 months (− 37.2 µm,* p* < 0.001) after the first injection, and then gradually increased up to 1 year (− 24.9 µm,* p* < 0.001). This could be explained by the treat-and-extend protocol employed in this study. Tamashiro et al. reported significantly decreased SCT (− 12.1 µm,* p* = 0.039) in the switched group at the 3-month visit from baseline^26^. Another Japanese study (− 45 µm,* p* < 0.01), which only targeted treatment-naïve eyes with PCV, found a greater reduction in SCT after 1 year^27^. A relatively high PCV ratio in this study could explain the difference with the results of other studies. In this study, the PCV rate was 45.7%, which is higher than that in other regions (e.g., 8.3% in Europe), similar to the prevalence reported in South Koreans in their early 70s^28,29^. The treatment response after switching to brolucizumab was not significantly associated with any of the three subtypes (*p* = 0.073). Regarding fluid status change after 1 year, the fluid was well-controlled for a longer time in PCV than in other subtypes.

The polypoidal lesion (sharply peaked PED) in PCV and prechoroidal cleft correlated with disease activity in neovascular AMD^30,31^. The presence of these structural OCT biomarkers was significantly associated with treatment response after switching to brolucizumab. A good response was observed in the case of disease activity, meaning that a good response should also be expected in treatment-naïve patients. In this study, regression of sharply peaked PED (16 of 31 eyes, 51.6%) was observed after two injections. In other studies reporting the short-term outcomes of brolucizumab for PCV, the regression rates of polypoidal lesions based on ICGA after 3 months were 78.9% (15 of 19 eyes) and 78.6% (11 of 14 eyes)^32,33^. Unlike previous studies, this study used non-ICGA-based criteria for diagnosing polypoidal lesions^34^.

The prechoroidal cleft, which refers to a hypo-reflective pocket of fluid located between Bruch’s membrane and the hyper-reflective material within the PED, is known to be associated with poor vision in the long term^35^. Viggiano et al.^8^ reported on the anatomical impact of brolucizumab on the choroidal layer. In this study, the presence of the prechoroidal cleft was found to be associated with a good response following the switch to brolucizumab, suggesting that brolucizumab has a comparable impact on the choroidal layer when compared with conventional anti-VEGF agents.

In this study, seven patients (8.6%) had intraocular inflammation (IOI) and one patient (1.2%) had retinal vascular occlusion, which is higher than in previous studies. The BRAILLE, BREW, and REBA studies reported no cases of IOI or vascular occlusion^19,20,22^. The number of prior anti-VEGF injections, the injection interval, and the treatment response to brolucizumab were not significantly correlated with the development of adverse events. Additionally, baseline anatomical measurements, such as CRT, SCT, and PED, were also not associated with adverse events. No serious complications were observed after switching to brolucizumab.

In the first month after the initial injection, all patients showed significant anatomical improvement (*p* < 0.001). In the poor-response group, anatomic outcomes worsened within 2 months after injection. Structural changes at 2 months, particularly a thickening of the subfoveal choroid and PED may predict a poor response, even if there is early improvement after the initial injection. Most of the polypoidal lesions were early regressed within two injections. If the early response after switching to brolucizumab is not good, early replacement may be considered.

Limitations in this study may have influenced the conclusions. First, the retrospective nature of this design study limits detailed observations of active lesions. Second, the inclusion of various anti-VEGF agents prior to the switch could introduce bias and restrict the applicability of the findings. Therefore, conducting further studies with a larger patient population and categorizing patients based on specific agents is necessary to obtain more conclusive results. Quantification of fluid status should also be supplemented. There may also be errors caused by approximation when analyzing with converted ETDRS acuity.

In conclusion, brolucizumab is effective in patients with neovascular AMD that are refractory to previous anti-VEGF treatment. Most switch patients achieved visual gain and anatomical improvements that were maintained until the final follow-up with a low treatment burden. The identified OCT biomarkers may be predictors of treatment response and may assist in selecting appropriate therapeutic strategies.

## Data Availability

The datasets analyzed during the current study are available from the corresponding author on reasonable request.
